# Disruption of glial cell development by Zika virus contributes to severe microcephalic newborn mice

**DOI:** 10.1038/s41421-018-0042-1

**Published:** 2018-07-31

**Authors:** Cui Li, Qin Wang, Yisheng Jiang, Qing Ye, Dan Xu, Fei Gao, Jesse W. Xu, Ruoke Wang, Xingliang Zhu, Lei Shi, Lei Yu, Fuchun Zhang, Weixiang Guo, Linqi Zhang, Cheng-Feng Qin, Zhiheng Xu

**Affiliations:** 1 State Key Laboratory of Molecular Developmental Biology, CAS Center for Excellence in Brain Science and Intelligence Technology, Institute of Genetics and Developmental Biology, Chinese Academy of Sciences, Beijing, 100071 China; 2 University of Chinese Academy of Sciences, Beijing, 100101 China; 3 Department of Virology, State Key Laboratory of Pathogen and Biosecurity, Beijing Institute of Microbiology and Epidemiology, Beijing, 100071 China; 4 Institute of Life Sciences, Fuzhou University, Fuzhou, 350116 China; 5 Comprehensive AIDS Research Center, Collaborative Innovation Center for Diagnosis & Treatment of Infectious Diseases, Department of Basic Medical Sciences, School of Medicine, Tsinghua University, Beijing, 100084 China; 6 Department of Bioengineering, School of Engineering and Applied Science, University of Pennsylvania, Philadelphia, PA 19104 USA; 7 Guangzhou Eighth People’s Hospital, Guangzhou Medical University, Guangzhou, 510060 China; 8 Parkinson’s Disease Center, Beijing Institute for Brain Disorders, Beijing, 100101 China

## Abstract

The causal link between Zika virus (ZIKV) infection and microcephaly has raised alarm worldwide. Microglial hyperplasia, reactive gliosis, and myelination delay have been reported in ZIKV-infected microcephalic fetuses. However, whether and how ZIKV infection affects glial cell development remain unclear. Here we show that ZIKV infection of embryos at the later stage of development causes severe microcephaly after birth. ZIKV infects the glial progenitors during brain development. Specifically, ZIKV infection disturbs the proliferation and differentiation of the oligodendrocyte progenitor cells and leads to the abolishment of oligodendrocyte development. More importantly, a single intraperitoneal injection of pregnant mice with a human monoclonal neutralizing antibody provides full protection against ZIKV infection and its associated damages in the developing fetuses. Our results not only provide more insights into the pathogenesis of ZIKV infection, but also present a new model for the preclinical test of prophylactic and therapeutic agents against ZIKV infection.

## Introduction

The world’s attention has been drawn to a global Zika virus (ZIKV) outbreak and its link with devastating cases of microcephaly. The Brazilian Ministry of Health reported a 20-fold increase in cases of neonatal microcephaly, which corresponds geographically and temporally to the ZIKV outbreak in November 2015^1^. A causal link between ZIKV infection and microcephaly or fetal death was confirmed recently by the presence of microcephaly and other brain abnormalities in the pups of mice infected with ZIKV^2–5^.

Disturbance of the proper proliferation/self-renewal and differentiation of neural progenitor cells (NPCs), as well as neuronal migration and maturation, may lead to developmental brain disorders including microcephaly^6–9^. ZIKV readily infects NPCs and cerebral organoids in culture and in mouse brains^2,3,5,10–13^. The infection results in dysregulation of NPC proliferation, differentiation, and neuronal cell death. It has been proposed that ZIKV infects NPCs to cause immune responses and aberrant gene expression related to NPC development, triggering cell death and eventually leading to microcephaly^3,5,13^.

Recent studies have begun to expand from the neuron-specific analysis into additional cellular targets for ZIKV infection such as the glial cells^14–16^. Glial cells are recognized as critical players in brain physiology, metabolism, development, and neurological diseases^17–23^. Mammalian brains have been reported to be comprised of 50–90% glial cells, including macroglial cells such as oligodendrocytes, astrocytes, and microglia^17–20,23^. Compared to neurons, glial cells develop during the late stage of brain development and after birth, and represent half or more of the cells in the brain (up to 90% in specific parts of the human brain)^18,20^. Most cerebral cortex glial cells are oligodendrocytes (75.6%) and the rest are primarily astrocytes (17.3%) and microglia (6.5%)^24^. Therefore, the proper development of glial cells should also be critical for normal brain size and function. More importantly, intrauterine or congenital ZIKV infection can lead to reactive gliosis, microglial hyperplasia, corpus callosum hypoplasia, and delayed myelination^25–27^. However, whether and how the glial precursors are affected by ZIKV infection are not clear. Here we used contemporary Asian ZIKV strains, which are able to infect NPCs in the embryonic mouse brains and lead to microcephaly^3,28,29^ to establish a new fetal brain infection model in order to investigate whether ZIKV affects the development of glial cells in neonatal mice.

Currently, no vaccines or therapeutics are available against ZIKV infection in patients. However, several groups have successfully developed vaccines^30–33^ or isolated potent neutralizing monoclonal antibodies (mAbs)^34–36^, and tested for their prophylactic and therapeutic potential in various mouse models of ZIKV infection such as pregnant and non-pregnant mice with deficiency in type I IFN signaling. While selected mAbs demonstrated substantial levels of protection against infection and disease^34,35^, their protective effect against ZIKV infection in the fetal brains has not been examined. Here, we inspected the efficacy of two previously reported potent mAbs (ZK2B10^36^ and ZV-67 ^35^) in our new mouse microcephaly model. Our results demonstrated that a single intraperitoneal injection of pregnant mice with a low dose of human mAb provides full protection against ZIKV infection and its associated damages in the developmental brain.

## Results

### ZIKV infection at the later stage of development causes severe microcephaly

**Fig. 1:**
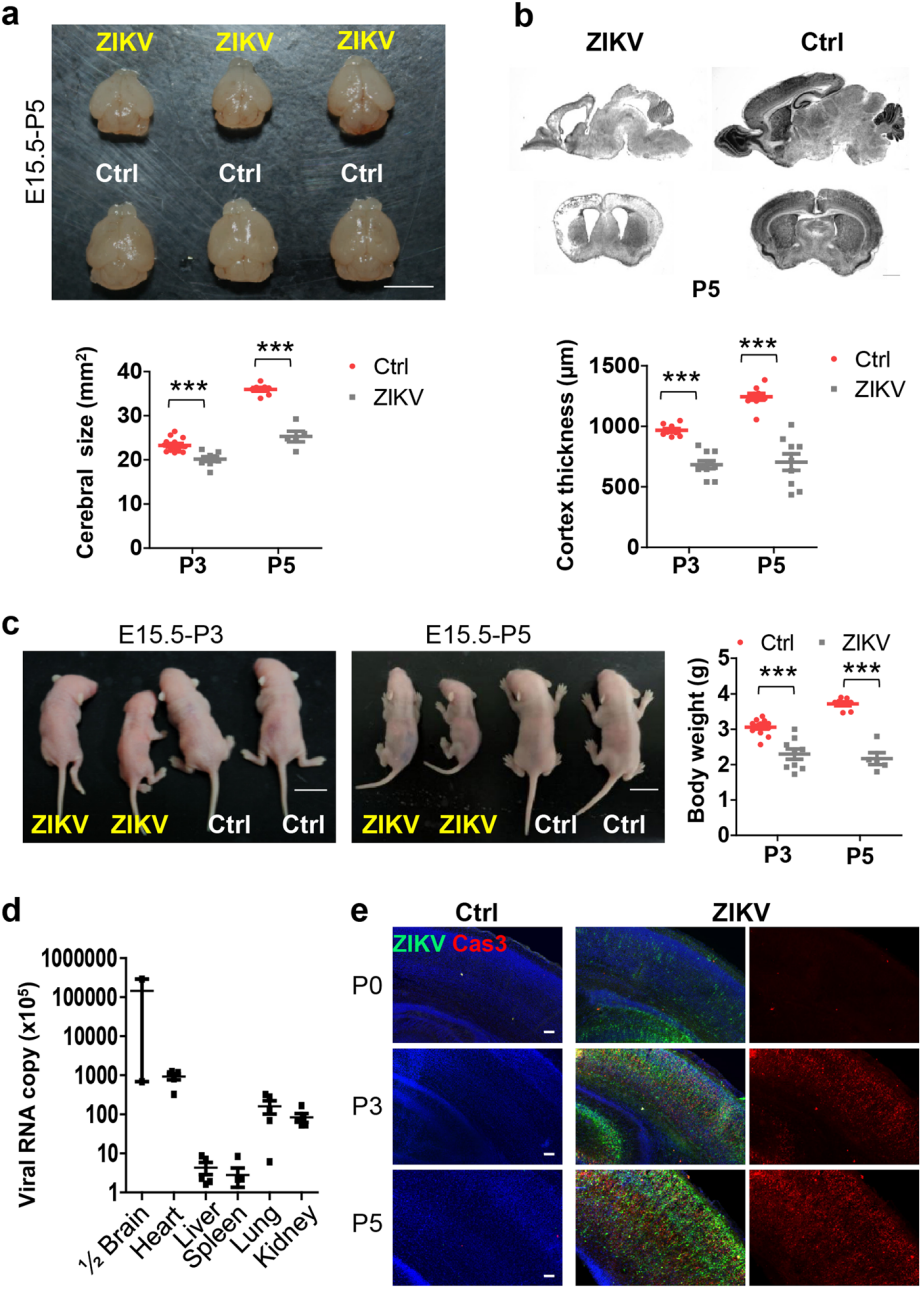
Zika virus infection at the late stage of development causes severe microcephaly. Embryonic brains were injected with ZIKV or medium in the lateral ventricular at E15.5 and inspected at P0, P3, or P5. **a** Neonate mouse brains of mock infected (Ctrl) and ZIKV infected (ZIKV) at P5. Right panel: quantification of cerebral size. P3: Ctrl *n* = 14, ZIKV *n* = 9, *P* = 0.0001; P5: Ctrl *n* = 8, ZIKV *n* = 5, *P* = 7 × 10^−7^. **b** Nissl staining of the coronal and sagittal brain slices at P5. Right panel: quantification of cortex thickness. P3: *n* = 10/4, *P* = 2 × 10^−7^; P5: Ctrl *n* = 10/3, ZIKV *n* = 9/3, *P* = 6 × 10^−7^. *n*: number of slices/different brains. **c** Images of neonatal mice at P3 and P5. Right panel: quantification of body weight. P3: Ctrl *n* = 14, ZIKV *n* = 9, *P* = 1 × 10^−5^; P5: Ctrl *n* = 8, ZIKV *n* = 5, *P* = 5 × 10^−7^. **d** Viral RNA copies in different tissues were determined by real-time PCR at P5. 1/2 Brains were from the injected side *n* = 2; other organs *n* = 5. **e** Images of coronal sections stained with antibodies for the activated form of caspase-3 (Cas3), ZIKV, and 4′,6-diamidino-2-phenylindole (DAPI). All data are mean ± SEM. ns: no significant, *P < 0.05, **P < 0.01, ***P < 0.001. Scale bars: 5 mm (**a**), 1 mm (**b**), 1 cm (**c**), 80 μm (**e**)

ZIKV infection at the second trimester of pregnancy in women, which is comparable with the late stage in mice, has been reported to cause microcephalic children^37^. Based on the finding of ZIKV in the aborted fetus brain, a mouse microcephaly model was established^3^. However, littermates infected at embryonic day 13.5 (E13.5) died soon after birth which prevents the analysis of brain development at postnatal stages. To investigate the consequences of embryonic ZIKV infection after birth, we tried to improve the existing ZIKV brain infection model. Specifically, the contemporary ZIKV strain, SZ01 or culture medium was injected into the lateral ventricle of E15.5 littermate brains. Infected brains were substantially smaller in size, thinner in cortex, and larger in lateral ventricle compared to that of uninfected mice inspected at postnatal day 3 (P3) and P5 (Fig. 1a, b and Supplementary Fig.1c–d). Brain size and cortex were not affected very significantly by ZIKV infection at P0 (Supplementary Fig. S1a–b). Interestingly, we found reduced body size in infected P3 and P5 pups (Fig. 1c), reminiscent of those infected children^37^. This implicates that the body might also be infected by ZIKV. A large amount of viral RNA copies was detected by quantitative real-time PCR at P5 in different organs, including the heart, lung, kidney, liver, and spleen, in addition to the brain (Fig. 1d). Similar to some ZIKV-infected neonates in humans^37^, the infected littermates died about 5–9 days after birth (Supplementary Fig. S1e).

Increased cell death was associated with ZIKV infection and contributed to microcephaly^3,38^. To determine whether cell death contributes to the smaller size of infected neonatal brains, we inspected the littermate brains at P3 and P5. Compared to mock-infected brains, many cells in the cortex were strongly positive for the activated form of caspase-3 (Cas3) in the infected brains (Fig. 1e). Detailed analysis of the infected brains by immunocytochemistry indicated that not all of Cas3^+^ cells were positive for ZIKV, and the Cas3 signal could co-localize with markers for neuron, astrocyte, and microglia (Supplementary Fig. S2). Most of apoptotic cells were neurons and some of the dying cells were astrocytes. Co-localization of Cas3 with Iba-1 (a marker for microglia) signals might be due to the swallowing of dead cells by microglia.

### ZIKV infection leads to progressive activation of microglia and astrocyte

We went on to study which parts and what kinds of cells in the neonatal brains were infected by ZIKV using an immunocytochemistry approach. ZIKV-infected cells were detected in multiple areas in the brain including the cortex, hippocampus, striatum, thalamus, midbrain and cerebellum at P0, P3, or P5 (Supplementary Fig. S3a). Most ZIKV-infected cells were positive for the neuronal marker NeuN and the loss of neurons was detected as reported^39^. However, closer examination revealed that about 22% of the ZIKV-infected cells were negative for NeuN (Supplementary Fig. S3b and c). This suggests that ZIKV infects non-neuronal cells, very likely the glial cells, which compose 50–90% of the cells in mammalian brains^17–20,23^.

Microglial cells are specialized macrophages capable of phagocytosis^8,17^. To determine the effect of ZIKV infection on microglial cells, we stained littermate cortices infected or mock infected at E15.5 with Iba-1, a marker for microglia. While a weak signal of Iba-1 was detected in control cortices, there were many more cells strongly positive for Iba-1 in the infected P3 and P5 cortices (Supplementary Fig. S4a). The increase in the measured signal was due to the activation of microglia, as most of the Iba-1^+^ cells were also positive for CD68, a marker for phagolysosome and activated microglia (Supplementary Fig. S4b). In addition, ZIKV was found in the CD68-positive vesicles (Supplementary Fig. S4c).

Since 17.3% of the cerebral cortex glial cells are astrocytes^24^, we also inspected astrocytes in the infected brain. A strong GFAP signal (a marker for astrocytes) was detected at P3 and P5, but not at P0 (Supplementary Fig. S5a). This indicates that ZIKV induces the activation of astrocytes at the later stages of infection. Proliferation contributes to severe diffuse astrogliosis. To check whether the increased GFAP expression might be due to reactive astrocyte amplification, we co-stained BrdU and GFAP, GFAP and Ki67 in the infected brains. The results indicate that, at least, some of the reactive astrocytes are proliferating (Supplementary Fig. S5b).

### ZIKV infection disturbs the development of glial progenitor cells

**Fig. 2:**
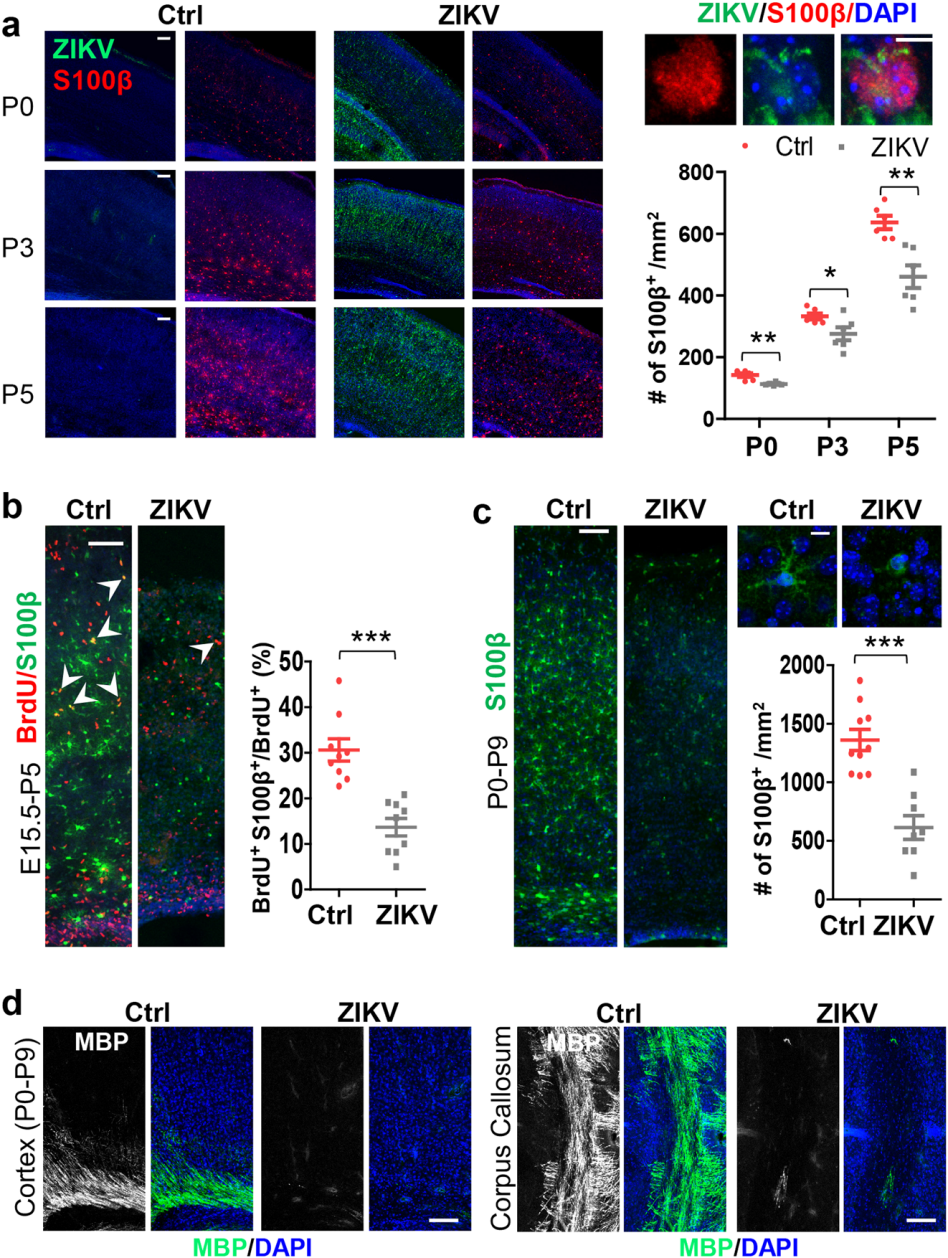
ZIKV infection disturbs the development of glial progenitor cells. **a** Coronal sections stained for calcium-binding protein β (S100β), ZIKV, and DAPI. Right panel: quantification of S100β^+^ cells. P0: *n* = 6/5, *P* = 0.001; P3: *n* = 6/5, *P* = 0.03; P5: *n* = 6/3, *P* = 0.002. **b** Cortices from P5 brains were stained for 5-bromo-2′-deoxyuridine (BrdU, labeled for 24 h) and S100β. Right panel: quantification of S100β and BrdU double-positive cells per total BrdU^+^ cells. *n* = 9/3, *P* = 5 × 10^−5^. **c** Cortices from P9 brains were stained for S100β. Right panel: quantification of S100β^+^ cells. Ctrl: *n* = 10/4, ZIKV: *n* = 8/3, *P* = 5 × 10^−5^. **d** Slices from P9 brains were stained for myelin basic protein (MBP). All data are mean ± SEM. *P < 0.05, **P < 0.01, ***P < 0.001. *n*: number of slices/different brains. Scale bars: 80 μm (**a**–**d**), 5 μm (**a**, **c**, right panels)

Glial cells play diverse and essential regulatory roles in the developing brains including neuronal survival^17–20,23^. We went on to determine whether ZIKV infection could affect the development of glial cells. Cortices were examined for S100β, a marker for astrocyte and oligodendrocyte progenitor cells (OPC)^40–42^. S100β^+^ cells were detected in the control cortex at P0 and more at P3 and P5 (Fig. 2a). However, significantly fewer S100β^+^ cells were detected in the counterpart of infected brains. The infection of glial progenitor cells was confirmed by double staining with both S100β and ZIKV sera (Fig. 2a). In addition, dramatically fewer S100β^+^ cells were also detected in infected brains labeled by BrdU injection at E18.5 and inspected at P5 (Supplementary Fig. S6). To exclude the possibility that those cells might die from apoptosis during that period, we tried BrdU labeling again for 24 h. Compared with the controls, the number of cells positive for both S100β and BrdU also dropped substantially in the infected brains (Fig. 2b). Finally, we infected newborn littermates at P0 and inspected the brains at P9 and found that there were dramatically less S100β^+^ cells in the infected brains (Fig. 2c). These results indicate that ZIKV infection affects the development of glial progenitor cells.

Neurogenesis has been shown to be disrupted when the brain is infected by ZIKV during the early and middle stages of mouse brain development^3,43^. We inspected, in the current model (infection at E15.5), whether neurogenesis might be affected at E18.5 and P0 when neurogenesis begins to switch to gliogenesis^18,20^. As shown in Supplementary Fig. S7, there were no apparent differences in neuron number and layer thickness between the control and infected brains (Supplementary Fig. S7a and b). In addition, we labeled the proliferating cells one day before birth and detected no significant difference in cell proliferation (Ki67-positive cell) and cycle exit index between the two groups at P0 (Supplementary Fig. S7c). This indicates that ZIKV infection at E15.5 may not disrupt neurogenesis significantly in the cortex.

### ZIKV infection disrupts oligodendrocyte development

**Fig. 3:**
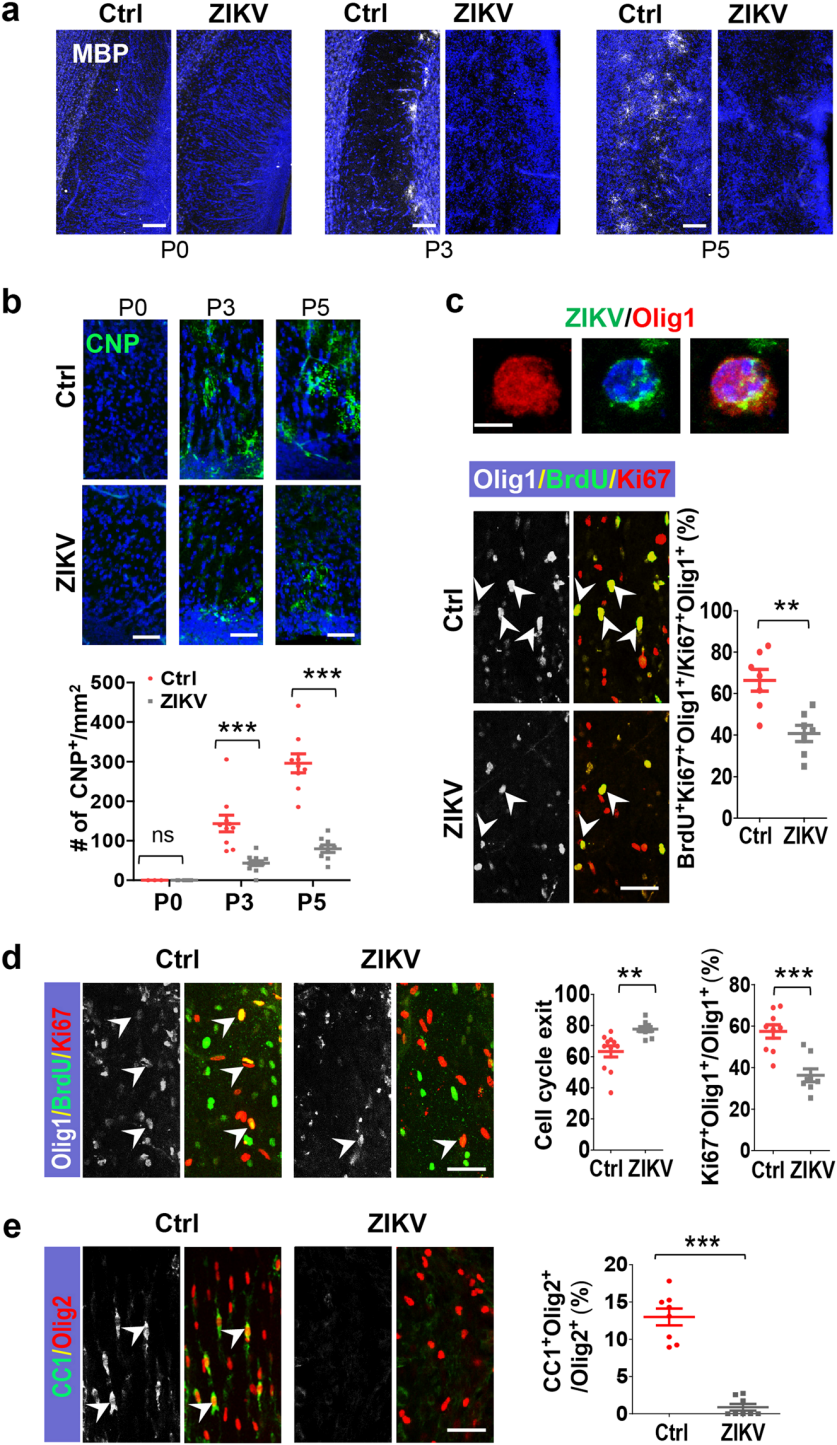
Oligodendrocyte development is disrupted by ZIKV infection. Sections of neonatal brains were infected or mock infected at E15.5 and inspected at P0, P3, and P5. **a** Sagittal sections of corpus callosum stained for MBP and DAPI. **b** Sagittal sections of corpus callosum stained for 2′,3′-cyclic-nucleotide 3′-phosphodiesterase (CNP) and DAPI, and quantified for CNP^+^ cells. P0: Ctrl *n* = 3/3, ZIKV *n* = 4/3; P3: Ctrl *n* = 10/4, ZIKV *n* = 12/4, *P* = 0.0001; P5: Ctrl *n* = 9/3, ZIKV *n* = 9/3, *P* = 3 × 10^−7^. **c** Upper panel: P5 brain staining for oligodendrocyte transcription factor 1 (Olig1), ZIKV, and DAPI. Lower panels: corpus callosum sections from P3 brain stained for Olig1, BrdU (labeled for 1 h), and Ki67, and quantified for Olig1^+^BrdU^+^Ki67^+^ cells per total Ki67^+^Olig1^+^ cells. *n* = 7/4, *P* = 0.002. **d** Corpus callosum sections from P3 brains were stained for Olig1, BrdU (labeled for 24 h), and Ki67. Right panels: quantification of cell cycle exit: BrdU^+^Olig1^+^Ki67^−^ cells per total BrdU^+^Olig1^+^ cells. Ctrl: *n* = 11/3, ZIKV: *n* = 9/3, *P* = 0.003; quantification of Ki67^+^Olig1^+^ cells per total Olig1^+^ cells. Ctrl: *n* = 9/3, ZIKV: *n* = 8/3, *P* = 0.0003. **e** Images of corpus callosum regions from P5 were co-stained for adenomatous polyposis coli clone CC1 and oligodendrocyte transcription factor 2 (Olig2). Right panel: quantification of CC1 and Olig2 double-positive cells per total Olig2^+^ cells. *n* = 8/3, *P* = 9.8 × 10^−8^. All data are mean ± SEM. **P < 0.01, ***P < 0.001. *n*: number of slices/different brains. Scale bars: 80 μm (**a**), 40 μm (**b**–**e**), 5 μm (**c**, upper panel)

The myelination of axons by oligodendrocytes forms an electrical insulator in vertebrates and represents a novel form of plasticity in the CNS^17,19,20,23^. Since 75.6% of cerebral cortex glial cells are oligodendrocytes^24^, we inspected the oligodendrocytes in P9 brains which were infected at P0. The signal for MBP, a marker for oligodendrocyte, was barely detectable in both the corpus callosum and cortex regions in the infected brains (Fig. 2d). This indicates that ZIKV infection leads to a loss of oligodendrocytes and/or defects in their development. We therefore investigated the development of oligodendrocytes in littermate cortices infected or mock infected at E15.5 after birth through staining for markers of oligodendrocyte lineages at different stages. MBP^+^ cells were detected in the white matter at P3 and much more at P5 in the control brains. Strikingly, no apparent MBP^+^ cells were detected in the infected brains (Fig. 3a). Similarly, the numbers of CNP^+^ cells (CNP is a marker for immature oligodendrocytes) were reduced very significantly in the P3 and P5 infected cortex (Fig. 3b).

We went on to inspect oligodendrocyte progenitor cells (OPC), which are positive for Olig1 and Olig2 at P0. No significant difference in the numbers of Olig1- and Olig2-positive cells was detected between mock and ZIKV-infected brains (Supplementary Fig. S8a). This suggests that Zika infection at E15.5 may not affect significantly the switch of radial glial cells to glial progenitor cells. However, we found OPC could be infected by ZIKV in the P5 cortex (Fig. 3c and Supplementary Fig. S8b). To determine whether the process of proliferation and differentiation of OPC were affected, littermates infected at E15.5 were labeled by BrdU injection 1 h before sacrifice at P3. Substantially fewer cells positive for both Olig1 and Ki67 (a marker for proliferative cells) were labeled by BrdU in infected brains (Fig. 3c). In addition, we injected BrdU at P2 and inspected at P3, and found that the number of cells positive for both Olig1 and Ki67 dropped significantly, accompanied by an increase in cell cycle exit by Olig1^+^ cells (Fig. 3d). Such a substantial drop of proliferating OPC was unlikely due to an evident increase of apoptosis (Supplementary Fig. S8c). Moreover, we performed double staining of the P5 corpus callosum regions for both Olig2 and CC1, which is expressed in oligodendrocytes but not in OPCs. The dramatic drop in number of cells positive for both Olig2 and CC1 relative to those for Olig2 (Fig. 3e) suggests that the differentiation of OPC was seriously affected.

The above results indicate that ZIKV infection disturbs OPC proliferation and differentiation, and leads to the complete disruption of oligodendrocyte development at the late stage of brain development.

### A human mAb provides full protection against ZIKV infection and its associated damages in the developmental brain

We previously isolated a panel of human mAbs targeting the envelope glycoprotein of ZIKV with distinct neutralizing activities from a ZIKV convalescent individual^36^. Among them, ZK2B10 was the most potent and provided an impressive level of protection against ZIKV infection^36^. We compared ZK2B10 with the ZIKV antiserum which was used for the detection of ZIKV previously^3,44,45^ and found that ZK2B10 was also very effective (Supplementary Fig. S9). A mouse mAb, ZV-67, was isolated previously from an infected mouse and was able to suppress ZIKV infection in mice^35^. We therefore tried to illustrate in more detail whether the two antibodies can prevent the disruption of glial cell development in ZIKV-infected brains.

**Fig. 4:**
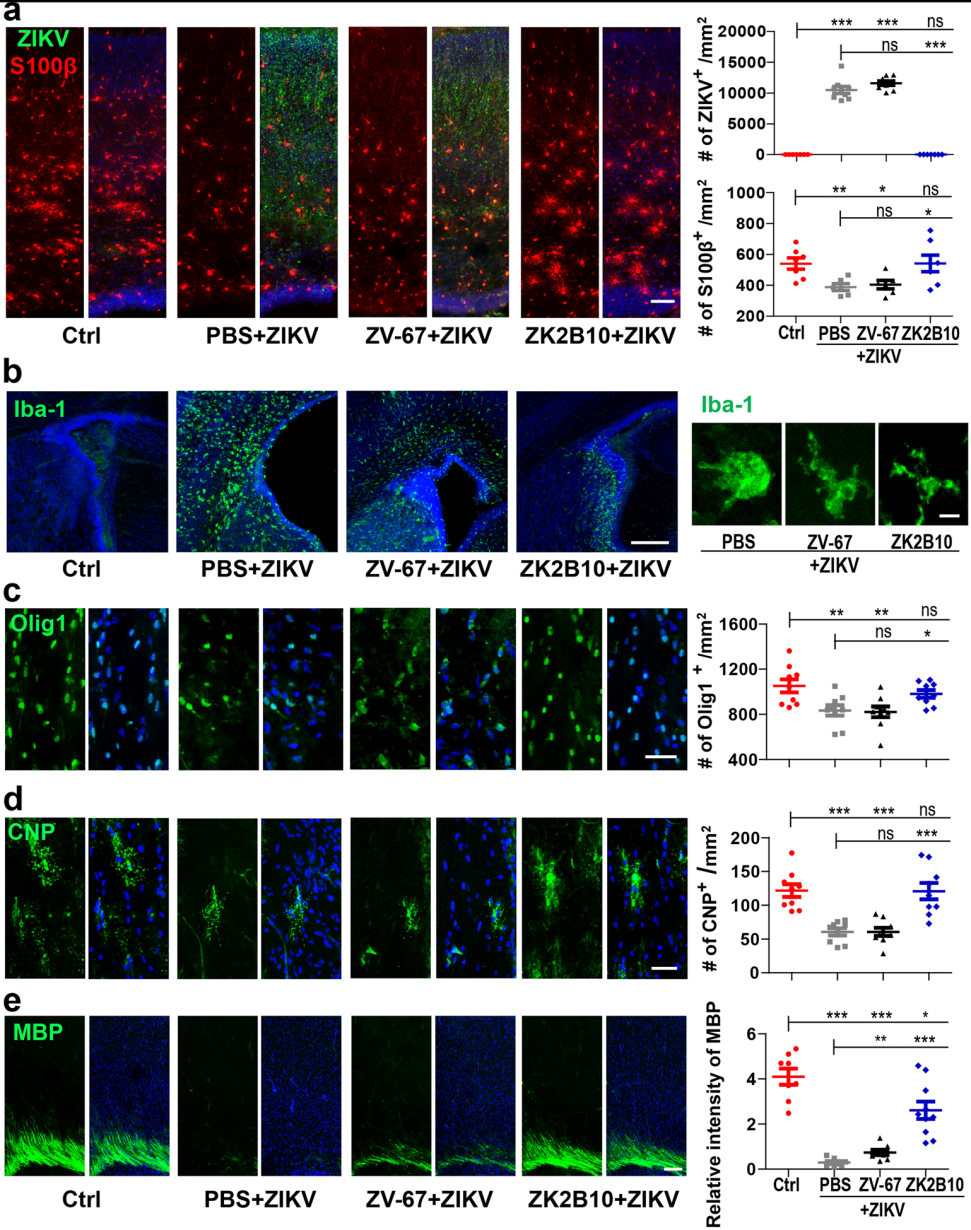
Human mAb provides full protection against ZIKV infection associated damages in the developmental brain. Sections of neonatal brains infected or mock infected at E15.5 were inspected at P3. Antibody or PBS was injected about 4–6 h before virus injection (**a**–**d**). **a** Sections of cortices stained for ZIKV, S100β, and DAPI. Right panels: quantification of ZIKV and S100β positive cells. ZIKV: Ctrl *n* = 7/4, PBS + ZIKV *n* = 9/4, ZV-67 + ZIKV *n* = 9/4, ZK2B10 + ZIKV *n* = 7/4. Ctrl & PBS + ZIKV: *P* = 1.7 × 10^−10^; Ctrl & ZV-67 + ZIKV: *P* = 7.2 × 10^−14^; Ctrl & ZK2B10 + ZIKV: *P* = 0.2221; PBS + ZIKV & ZV-67 + ZIKV: *P* = 0.1180; PBS + ZIKV & ZK2B10 + ZIKV: *P* = 1.7 × 10^−10^. S100 β: Ctrl *n* = 7/5, PBS + ZIKV *n* = 6/4, ZV-67 + ZIKV *n* = 6/4, ZK2B10 + ZIKV *n* = 7/4. Ctrl & PBS + ZIKV: *P* = 0.0059; Ctrl & ZV-67 + ZIKV: *P* = 0.0144; Ctrl & ZK2B10 + ZIKV: *P* = 0.2221; PBS + ZIKV & ZV-67 + ZIKV: *P* = 0.6523; PBS + ZIKV & ZK2B10 + ZIKV: *P* = 0293. **b** Sections were stained for ionized calcium-binding adapter molecule 1 (Iba-1) and DAPI. Enlarged representative images of Iba1^+^ cells are shown in the right panel. **c** Corpus callosum sections stained for Olig1 and DAPI. Right panel: quantification of Olig1^+^ cells. Ctrl *n* = 9/4, PBS + ZIKV *n* = 9/4, ZV-67 + ZIKV *n* = 9/4, ZK2B10 *n* = 9/4. Ctrl & PBS + ZIKV: *P* = 0.0097; Ctrl & ZV-67 + ZIKV: *P* = 0.0074; Ctrl & ZK2B10 + ZIKV: *P* = 0.3048; PBS + ZIKV & ZV-67 + ZIKV: *P* = 0.8436; PBS + ZIKV & ZK2B10 + ZIKV: *P* = 0.0206. **d** Corpus callosum sections were stained for CNPase. Right panels: quantification of CNPase^+^ cells. Ctrl *n* = 9/4, PBS + ZIKV *n* = 9/4, ZV-67 + ZIKV *n* = 9/4, ZK2B10 *n* = 9/4. Ctrl & PBS + ZIKV: *P* = 3.7 × 10^−5^; Ctrl & ZV-67 + ZIKV: *P* = 5.1 × 10^−5^; Ctrl & ZK2B10 + ZIKV: *P* = 0.9537; PBS + ZIKV & ZV-67 + ZIKV: *P* = 0.9867; PBS + ZIKV & ZK2B10 + ZIKV: *P* = 0.0003. **e** Antibody or PBS was injected about 0.5 h before virus injection at P0. Slices from P9 brains were stained for MBP. Right panel: quantification of the relative intensity of MBP. Ctrl *n* = 8/3, PBS + ZIKV *n* = 8/3, ZV-67 + ZIKV *n* = 8/3, ZK2B10 + ZIKV *n* = 10/3. Ctrl & PBS + ZIKV: *P* = 5.9 × 10^−8^; Ctrl & ZV-67 + ZIKV: *P* = 4.1 × 10^−7^; Ctrl & ZK2B10 + ZIKV: *P* = 0.0134; PBS + ZIKV & ZV-67 + ZIKV: *P* = 0.0048; PBS + ZIKV & ZK2B10 + ZIKV: *P* = 6.2 × 10^−5^. All data are mean ± SEM. ns: no significant, *P < 0.05, **P < 0.01, ***P < 0.001. *n*: number of slices/different brains. Scale bars: 80 μm (**a**), 300 μm (**b**), 40 μm (**c**, **d**), 300 μm (**e**), 10 μm (**b**, right panel)

For pretreatment, 5 mg/kg of ZK2B10, ZV-67, or PBS was intraperitoneally administered to pregnant mice at E15.5. About 4–6 h later, 350 PFU of ZIKV Asian strain GZ01, which was isolated from a patient returned from Venezuela in 2016^46^, was injected into the lateral ventricle of littermate embryonic brains. Neonatal cortices were inspected later at P3. Similar to the brains infected with SZ01 (Fig. 2a), a significant decrease in S100β^+^ cell density was detected in GZ01-infected littermate brains from pregnant mice pretreated with either PBS or ZV-67 (Fig. 4a). In contrast, no such decrease in S100β^+^ cell density was detected in littermates from those pretreated with ZK2B10 (Fig. 4a). Furthermore, immunohistochemical analysis showed that there were very few ZIKV-infected cells in the cortex pretreated with ZK2B10 compared with the substantially more infected cells in the PBS and ZV-67-treated groups (Fig. 4a). These results show clearly that ZK2B10 was more potent than ZV-67 in blocking ZIKV replication in vivo. In agreement with this observation, ZK2B10, but not ZV-67, was also able to suppress the activation of microglia (Iba-1^+^ cells) induced by GZ01 infection (Fig. 4b). Nevertheless, much fewer of the round and strong Iba-1^+^ cells were detected in the ZV-67- and ZK2B10-treatment groups compared to the untreated group (Fig. 4b). This suggests that ZV-67 still had some effect in blocking the activation of microglia.

Moreover, we observed that GZ01 infection also led to the decrease of OPC (Olig1^+^) and immature oligodendrocytes (CNP^+^) (Fig. 4c, d). These effects were blocked significantly by ZK2B10, but not by ZV-67. The antibodies ZK2B10 and ZV-67, themselves had no effect on the morphology of microglia and number of Olig1^+^ cells (Supplementary Fig. S10a and b). Finally, to inspect the effect of antibody treatment on the myelination of oligodendrocyte, we pretreated littermates with PBS or mAb half an hour before virus infection at P0 and inspected at P9. Oligodendrocytes (MBP^+^) were barely detectable in the cortex in GZ01-infected brains. ZK2B10 provided very significant protection in infected brains, while ZV-67 was only slightly effective (Fig. 4e). Meanwhile, the body weight, mortality rate, and loss of S100β^+^ cells were blocked significantly by ZK2B10, but not by ZV-67 (Supplementary Fig. S10c–e). These results indicate that oligodendrocyte development could be well protected by ZK2B10 mAb.

## Discussion

In this study, we improved the ZIKV brain infection model, which permitted the investigation of the consequences of embryonic ZIKV infection after birth till P9. In addition, we found that ZIKV in the brain can pass through the blood–brain barrier to replicate in different organs and systems in the fetus. This may account for the reduced body weight or even deaths of newborn babies delivered by ZIKV-infected women^1^. With this standardized new mouse model of microcephaly, we have discovered the progressive activation of microglia, astrogliosis, and the disruption of oligodendrocyte development by ZIKV infection, mimicking the symptoms of congenital ZIKV syndrome. Moreover, this model can be adopted for the systematic study of the prophylactic and therapeutic potential of antibodies, drugs, and vaccines against ZIKV infection.

Glial cells represent half or more of the cells in the brain (up to 90% in specific parts of the human brain)^18,20^. We demonstrate that ZIKV can directly infect different lineages of glial cells including oligodendrocytes, astrocytes, or their precursor cells, in addition to neurons and microglias at the late stage of brain development. We provide evidence that ZIKV infection leads to attenuated expansion of glial progenitors, especially oligodendrocyte precursor cells, through virally induced dysregulation of their proliferation and differentiation. Together with ZIKV’s effects on NPC development shown previously in the earlier stages of brain development^3^ and neuronal cell death in the current model, these effects would account for microcephaly in newborn infants. Thus, attention should be paid not only to NPCs and neurons, but also to glial cells and their precursor cells regarding their roles in ZIKV-associated brain pathogenesis.

Disruption of neurogenesis by ZIKV infection has previously been shown during early and middle stages of brain development^3,43^. We inspected, in the current model (infection at E15.5), whether neurogenesis might be affected around birth when neurogenesis begins to switch to gliogenesis^18,20^. No apparent difference in neuronal numbers or cortical layer thickness between the control and infected brains was detected at P0. Similarly, no significant differences in cell proliferation and cycle exit index were detected in the cortex. This indicates that neurogenesis may not be disrupted. Discrepancy between this study and the previous studies is very likely due to the reason that we injected tiny amounts of ZIKV (650 PFU) at E15.5 in order to mimic the pathogenesis of ZIKV infection in pregnant women. The virus would take several days (until birth) to replicate and infect most of the progenitors. Therefore, ZIKV infection may not affect the development of radial glia cells and neurogenesis very significantly before birth, but affect gliogenesis after birth instead in the current model.

Remarkably, we have shown that a single intraperitoneal injection of a low dose of human mAb, ZK2B10 (5 mg/kg), into pregnant mice provided the developing brains of the litter full protection against ZIKV infection as well as against its associated disruption of glial cell development. This is in support of our previous study that antibodies were able to cross the placental barrier as well as the primitive blood–brain barrier of the developing brain^43^. Similar to convalescent serum, no apparent detrimental effect of ZK2B10 or ZV-67 has been found in the fetal brain, including glial cell development as well as microglial activation (Supplementary Fig. S10a and b). Intriguingly, we found that mouse mAb ZV-67, which also targets the ZIKV E protein DIII^35^, could not provide protection as well as ZK2B10, although it could partially suppress the activation of microglial and loss of oligodendrocytes. Nevertheless, we have observed an increase of body weight of infected immunocompetent neonates by ZV-67 (Supplementary Fig. S10c). The apparent discrepancy is likely due to the following possibilities. One possibility is that ZK2B10 may have much higher neutralizing activity toward ZIKV than ZV-67. A low dosage of ZV-67 (5 μg/g), compared to a dosage of 250 μg/young mouse^35^, could not provide full protection. Another possibility could be that different models were adopted: fetal brain infection model vs. infection by subcutaneous route in mice with deficiency in type I IFN signaling^35^. The last reasonable possibility is that different strains were used: a strain from Venezuela vs. an African ZIKV strain^35^. Future studies would be warranted to verify these possibilities.

**Fig. 5:**
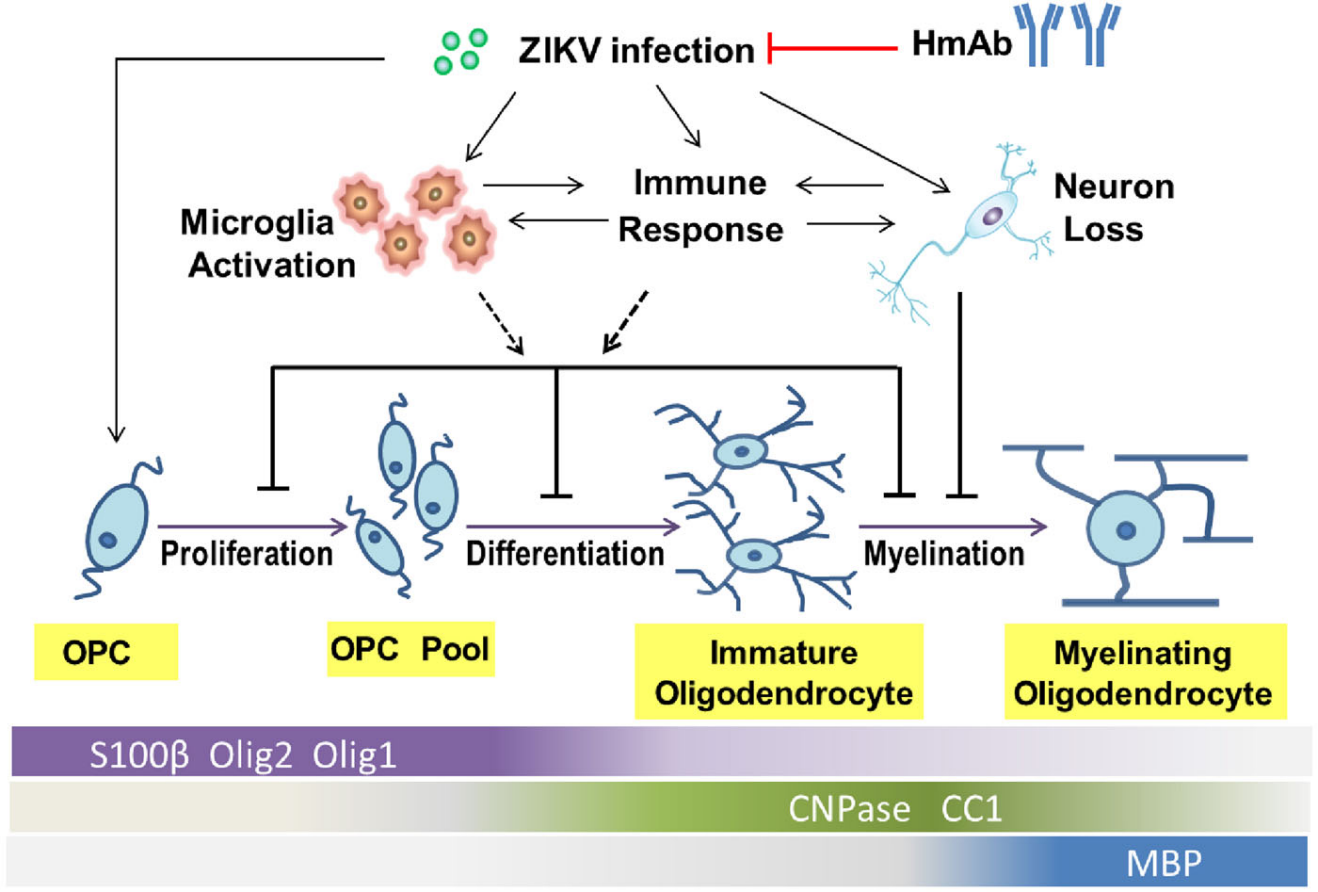
A model for ZIKV infection in the disturbance of oligodendrocyte development and the cause of microcephaly. ZIKV infection induces immune response which would lead to the activation of microglia and neuronal death. Together, they affect directly or indirectly the different stages of oligodendrocyte development, including proliferation and differentiation of oligodendrocyte precursor cells, and the myelination process. Additionally, human monoclonal antibody provides full protection against Zika virus infection in the developing fetuses

In summary, as depicted in Fig. 5, our findings indicate that ZIKV infection deregulates the expression of many genes involved in glial cell development, especially oligodendrogenesis. Meanwhile, ZIKV infection-induced immune responses, including the production of cytokines, would lead to the activation of microglia and astrocytes, as well as neuronal loss, which may affect the different stages of oligodendrocyte development indirectly^47^. More importantly, we provide a new model which is fit for the preclinical test of the prophylactic and therapeutic potential of agents against ZIKV infection.

## Methods

### Animal and ZIKV infection

The ICR pregnant mice were bought from Beijing Vital River Laboratory Animal Technology Co., Ltd. The experimental procedures were performed according to protocols approved by the Institutional Animal Care and Use Committee at Beijing Institute of Microbiology and Epidemiology. All the mice were reared on a 12/12 light/dark cycle. Fewer than five adult mice were housed in one cage and pregnant mice were kept alone before delivery.

One microliter of ZIKV SZ01 (GenBank accession no: KU866423) virus stock (6.5 × 10^5^ PFU/ml)^48^ or culture medium (RPMI medium 1640 basic + 2% FBS) was injected into one side of the lateral ventricle of the embryonic day 15.5 ICR mouse brains. ZIKV was injected as described previously^3^. For each pregnant dam, we injected virus or medium at the same side of fetal brains and inspected the neonate mice at P0, P3, or P5. For postnatal infection, we injected 100 PFU ZIKV or culture medium into the lambda point at P0, and analyzed at P9.

In mAb protection experiment, ZIKV GZ01 (Genbank no: KU820898, 350 PFU) was replaced for infection at E15.5. About 5 mg/kg of ZV-67, ZK2B10, or PBS as control, was intraperitoneally into pregnant mice 4–6 h before virus injection. Preparation of mAbs was described previously^36^. About 10 mg/kg of ZV-67 or ZK2B10 were used for postnatal infection about 30 min before injection of 100 PFU GZ01 into the lambda point at P0.

### Immunohistochemistry and antibodies

For cryosections, tissues were fixed in 4% PFA, dehydrated in 30% sucrose, and frozen in TFM (tissue freezing medium). Sections (thickness: 40 μm) were immunostained by blocking at room temperature (RT) for 1 h, first antibody at 4 °C for one night, PBST wash three times, followed by secondary antibody at RT for 1 h, and PBST wash three times. For CNPase staining, antigen retrieval was performed before blocking. Sections were incubating in 0.1 M trisodium citrate solution and microwaving at high level for 5 min, followed by low heat in the oven for another 15 min, and then natural cooling to RT.

The antibodies used for immunostaining were BrdU (Abcam, ab6326, 1:500), Activated-caspase-3 (Abcam, ab2302, 1:1000), NeuN (Abcam, ab104224, 1:1000), GFAP (DAKO, Z0334, 1:1000), CD68 (Abcam, ab125212, 1:1000), Iba-1 (Abcam, ab5076, 1:1000), S100β (DAKO, A5110, 1:1000), CNPase (Abcam, ab6319, 1:200), Olig1 (Chemicon, MAB5540, 1:250), MBP (Abcam, ab62631, 1:300), and Ki67 (Abcam, ab15580, 1:1000), Olig2 (Millipore, ab9610, 1:1000), CC1 (Calbiochem, OP80, 1:100). ZIKV antibody (1:100) was made from a convalescent patient serum. Nuclei were stained with DAPI (Invitrogen). BrdU was labeled for pregnant dams (50 mg/kg) or neonate (100 mg/kg) by intraperitoneal injection. Procedures for BrdU detection were described previously^38,49^. Slices were imaged on a LSM 700 (Carl Zeiss) confocal microscope.

### Nissl staining and confocal imaging

Brain slices were stained with 0.1% toluidine blue for 15 min, dehydrated in turn by 70%, 96%, 99% ethanol (45 s, twice for each). Finally, slices were hyalinized by xylene for more than 30 min.

Immunostaining slices were imaged on an LSM 700 (Carl Zeiss) confocal microscope, and the images were analyzed with Imaris, ImageJ, and Photoshop as described previously^38,49^.

### Statistical analysis

Image quantifications were performed by researchers blinded to the group allocation. All data were analyzed using Prism software (GraphPad) or Excel. Statistical evaluation was performed by Student’s unpaired *t-*test. Data are presented as mean ± standard error of the mean (s.e.m.). *P < 0.05, **P < 0.01, ***P < 0.001. All the representative images shown in the paper were the results of at least three independent experiments.

### Real-time qPCR

For viral RNA copies, total RNA were extracted from the injected side of P5 newborn mouse brains or other organs. Viral RNA copies were determined by real-time PCR, as described before^3^.

### Disclaimer

The data that support the findings of this study are available from the corresponding author upon reasonable request.

## Electronic supplementary material

Supplementary Information
