# Supplementary material for: Disruption of glial cell development by Zika virus contributes to severe microcephalic newborn mice
Source: Cell Discov. 2018 Jul 31;4:43. doi: 10.1038/s41421-018-0042-1 (PMC6066496; doi:10.1038/s41421-018-0042-1)
Supplement: Supplementary file 1 — Supplementary Information [file 41421_2018_42_MOESM1_ESM.pdf]

Figure S1

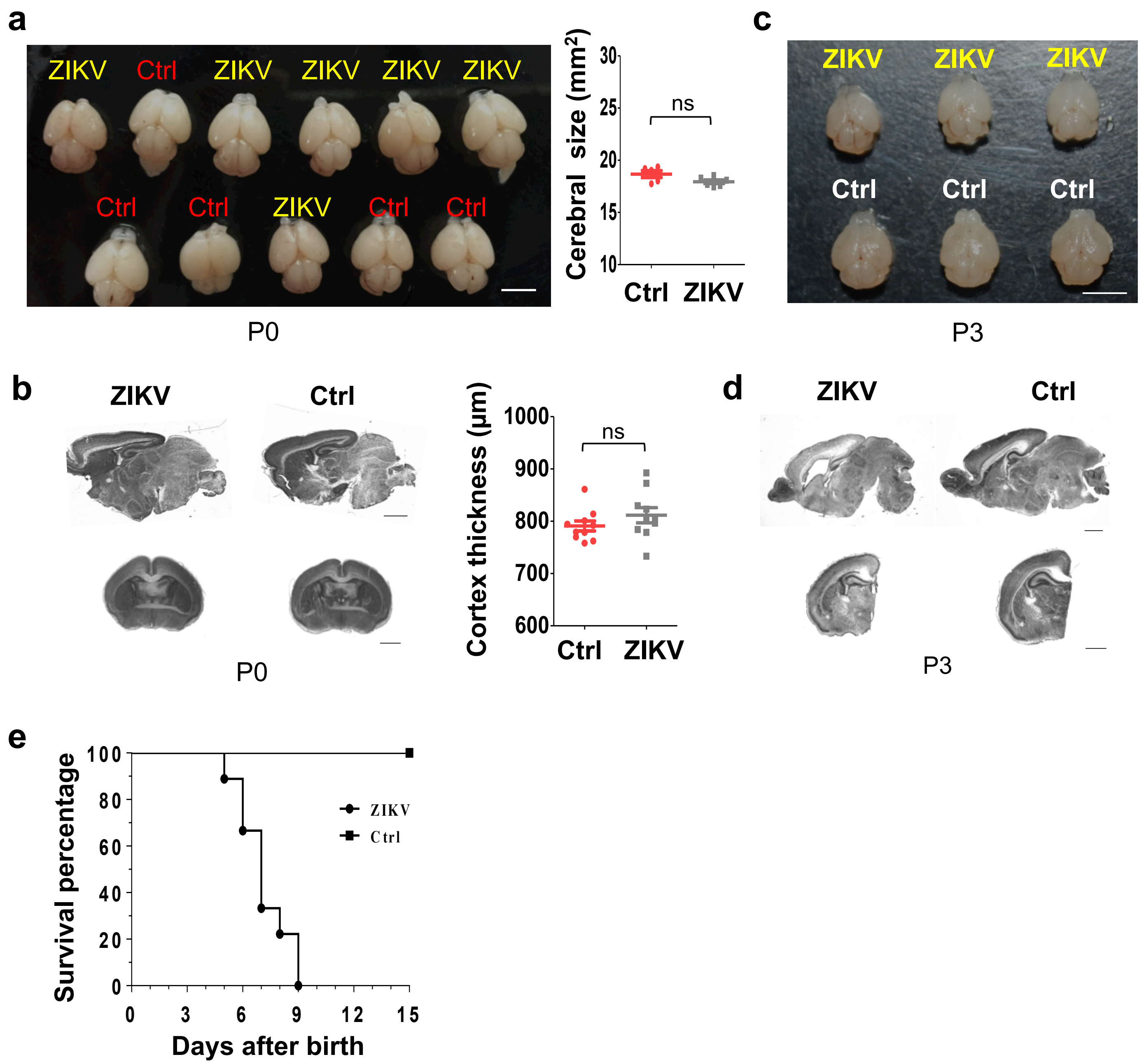

Figure S1. Embryonic littermate brains infected or mock infected at E15.5 and inspected after birth. (a) Images of mock and ZIKV infected brains at P0. Ctrl n=5, ZIKV n=6, p=0.0696. **(b)** Coronal and sagittal slices from control and ZIKV infected P0 brains by Nissl staining. Ctrl n=10, ZIKV n=10, p=0.2562. **(c)** Neonate mouse brains of mock-infected (Ctrl) and ZIKV infected (ZIKV) at P3. (**d)** Nissl staining of the coronal and sagittal brain slices at P3. **(e)** Two litters of new born pups were observed for their survival time. Ctrl n=10, ZIKV n=9. Data are mean ± SEM. ns: no significance. Scal bars: 5mm (a),1mm (b, d), 5 mm (c).

Figure S2

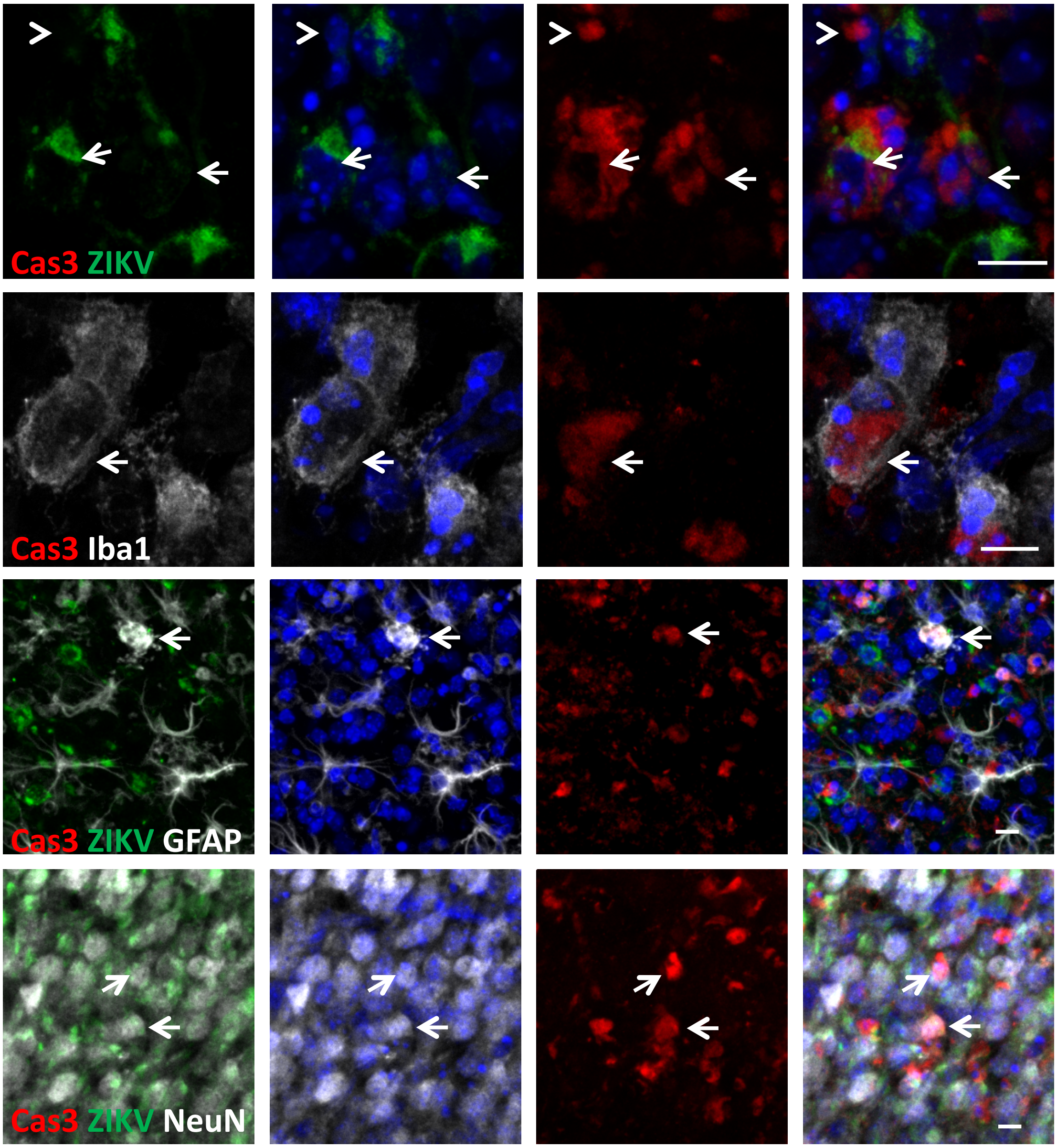

Figure S2. ZIKV infection leads to apoptosis of different cell types. Cortices sections from P3 or P5 were stained with Cas3, ZIKV antisera and different cell makers: Iba1, Glial fibrillary acidic protein (GFAP), and NeuN. Scale bar: 10μm.

Figure S3

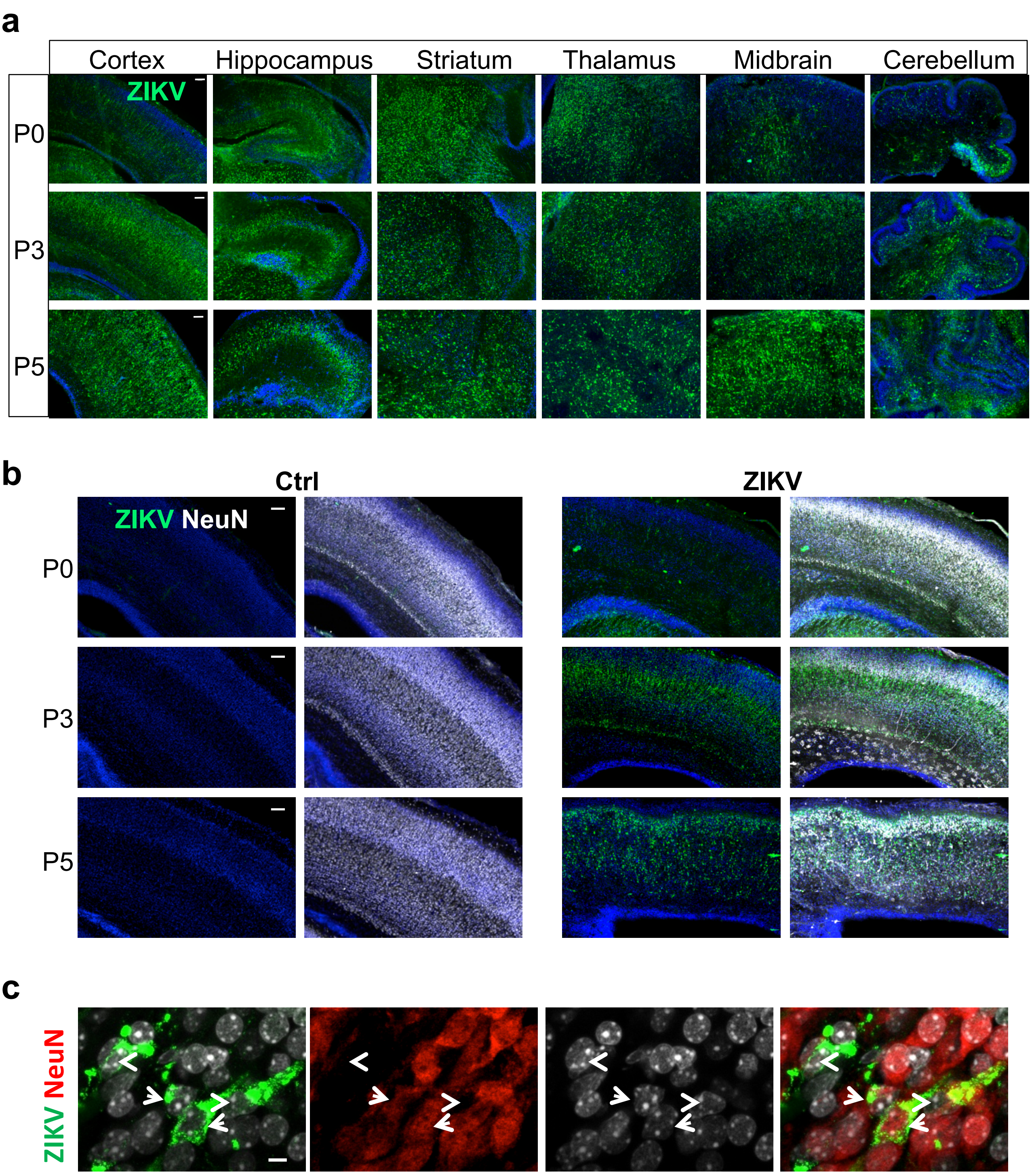

Figure S3. ZIKV infects different areas of the brain and leads to neuronal loss. Coronal sections of neonate pups infected or mock-infected at E15.5 and inspected at P0, P3, P5. **(a)** Images of brain sections stained with DAPI and ZIKV antiserum. **(b)** Cortices were stained with NeuN and ZIKV antisera. **(c)** Cortices from P0 were stained with NeuN and ZIKV antisera. Scale bars: 80µm (a,b), 5µm (c).

Figure S4

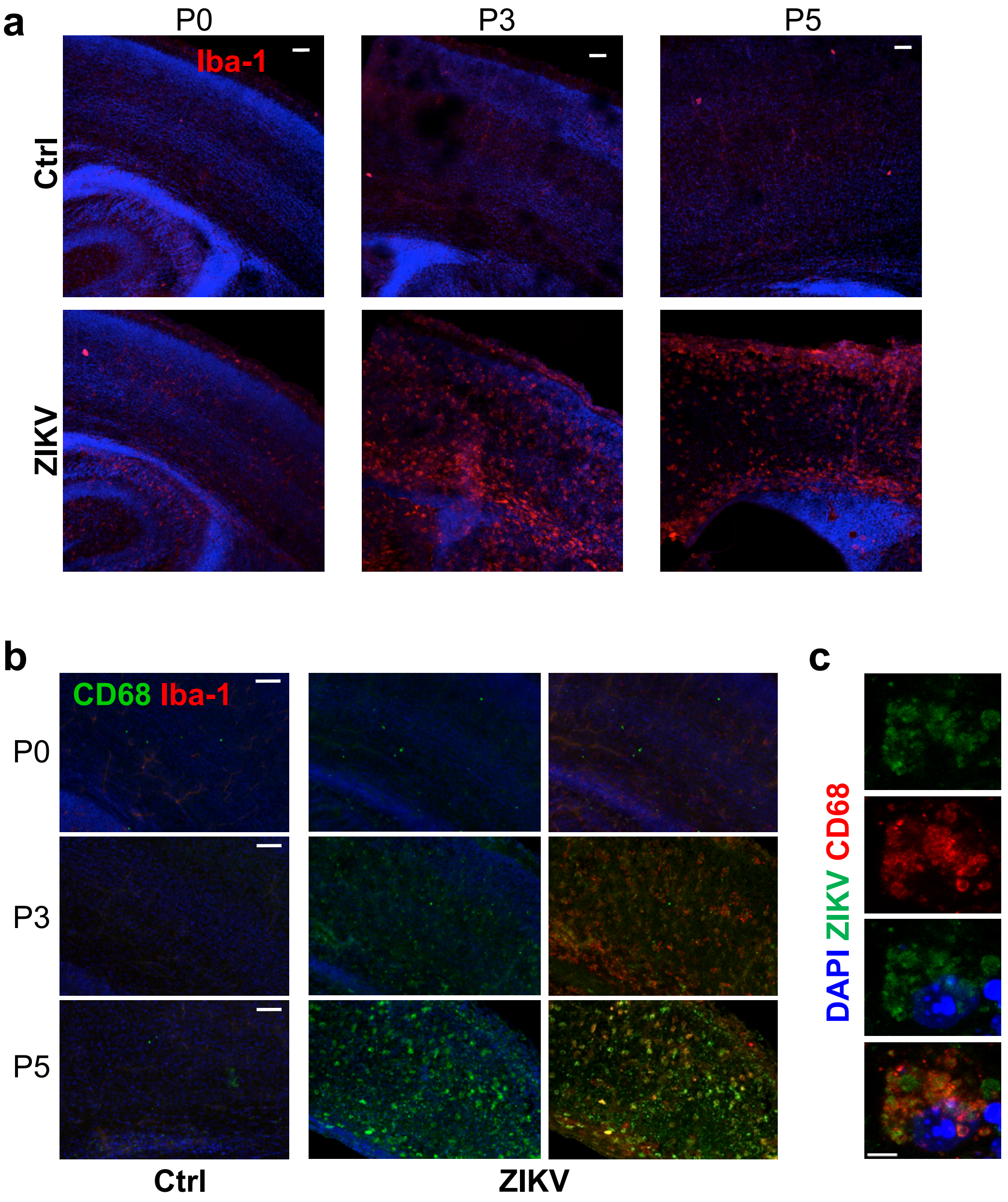

Figure S4. ZIKV infection leads to increase of microglia. Coronal sections of neonate pups infected or mock-infected at E15.5 and inspected at P0, P3, P5. **(a)** Coronal sections were stained for Iba-1 and DAPI. **(b)** Images of cortices were co-stained with antibodies for Iba-1, cluster of differentiation 68 (CD68) and DAPI. **(c)** Images of cortices were co-stained with antibodies for ZIKV and CD68. Scale bars: 80µm (a, b), 5µm (c).

Figure S5

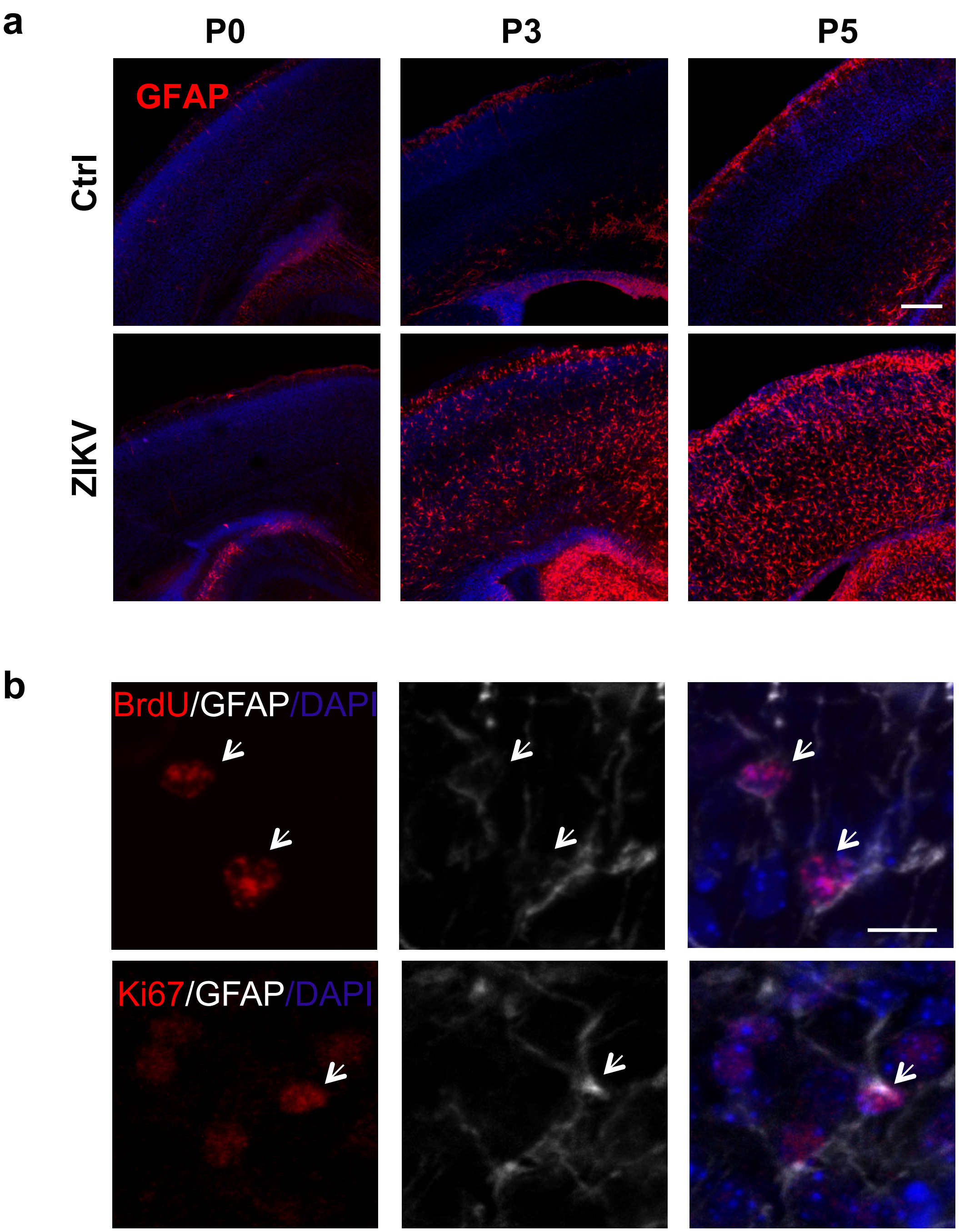

Figure S5. ZIKV infection leads to activation of astrocyte. (a) Coronal sections of neonate pups infected or mock-infected at E15.5 and inspected at P0, P3, P5. Brain sections were stained for GFAP and DAPI. **(b)** Sections from P3 infected mice (BrdU labeled for 24h) were stained for BrdU, GFAP or Ki67, GFAP. Scale bar: 200μm (a), 10 μm (b).

Figure S6

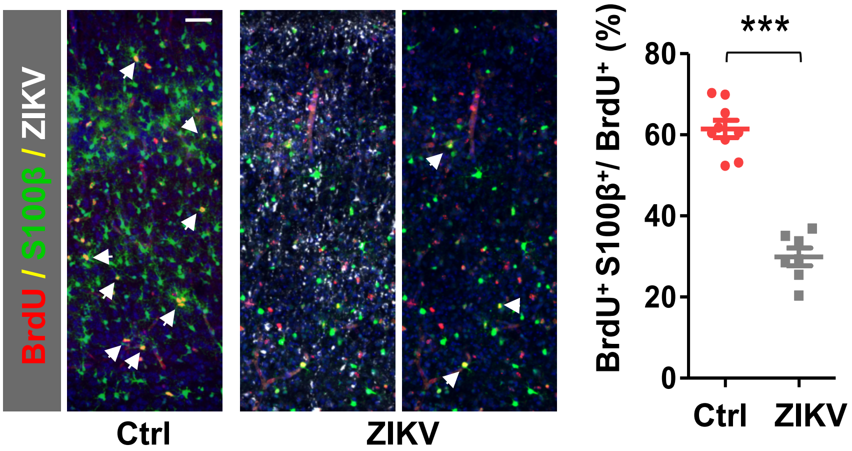

Figure S6. ZIKV infection leads to decrease of glial progenitor cells. Brains were infected or mock-infected at E15.5 and inspected at P5. Cortices were stained for BrdU (labeled at E18.5), S100β and ZIKV. Right panel: quantification of S100β and BrdU double positive cells per total $BrdU^+$ cells. Ctrl: n=9/3, ZIKV: n=7/3, $p=7\times10^{-8}$, n: number of slices/different brains. Data are mean ± SEM. ***p<0.001. Scale bars: 40μm.

Figure S7

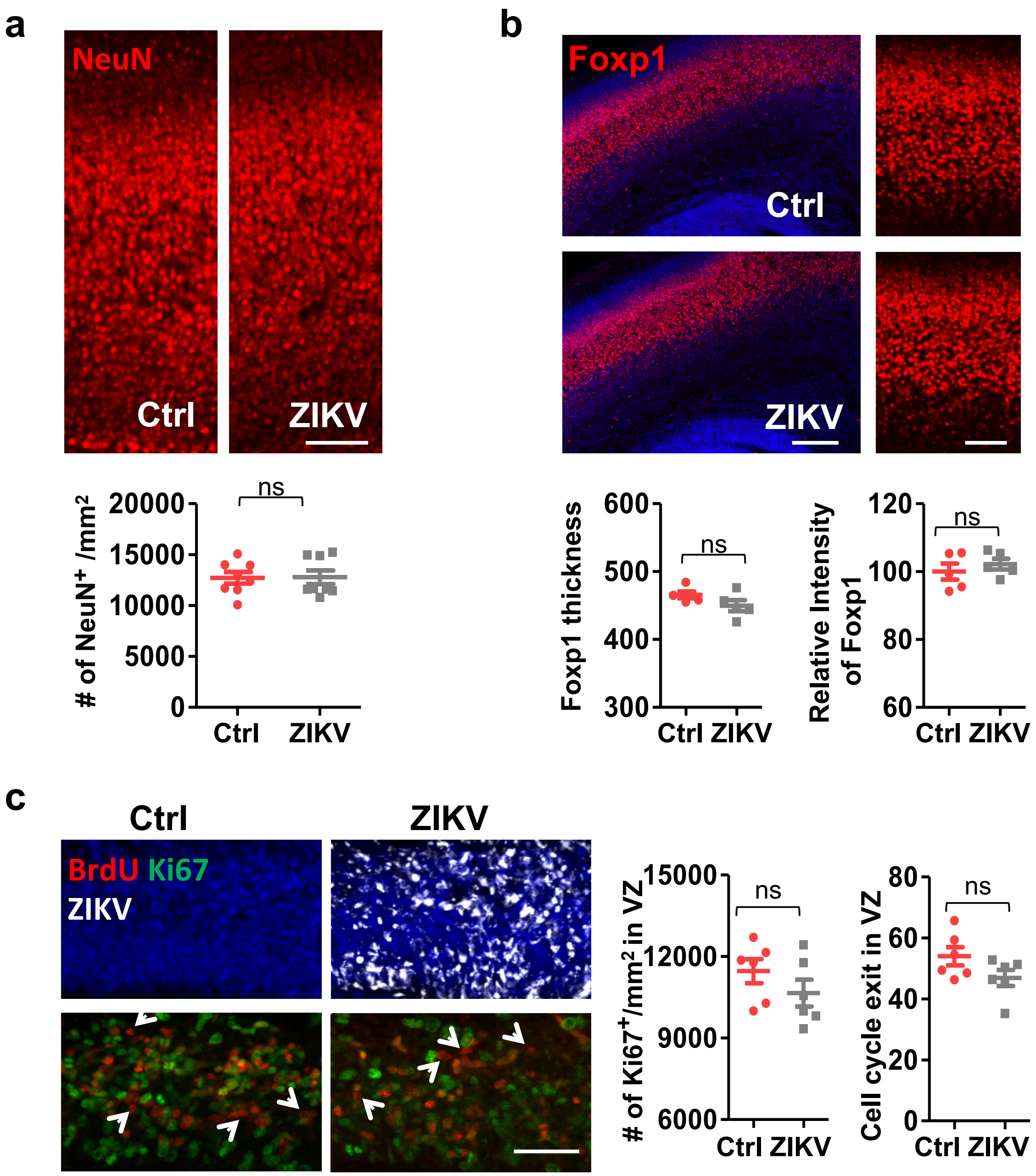

Figure S7. Neurogenesis is not affected very significantly by ZIKV infection at P0. Coronal sections of neonate pups infected or mock-infected at E15.5 and inspected at P0. **(a)** Coronal sections were stained for NeuN. Lower panel: quantification of NeuN+ cells. Ctrl n=8/3, ZIKV n=8/3, p=0.9424. **(b)** Coronal sections were stained for forkhead box protein P1 (Foxp1) and DAPI. Lower panel: quantification of Foxp1 thickness and relative intensity of Foxp1. Left: Ctrl n=5/3, ZIKV n=5/3, p=0.1402. Right: Ctrl n=5/3, ZIKV n=5/3, p=0.4620. **(c)** VZ were stained for BrdU, Ki67 and ZIKV antiserum. Right panel: quantification of Ki67+ cells and cell cycle exit. Left: Ctrl n=6/3, ZIKV n=6/3, p=0.2483. Right: Ctrl n=6/3, ZIKV n=6/3, p=0.1074. n: number of slices/different brains. Data are mean ± SEM. ns: no significance. Scale bars: 80μm (a, b right panel), 200μm (b left panel), 40μm (c).

Figure S8

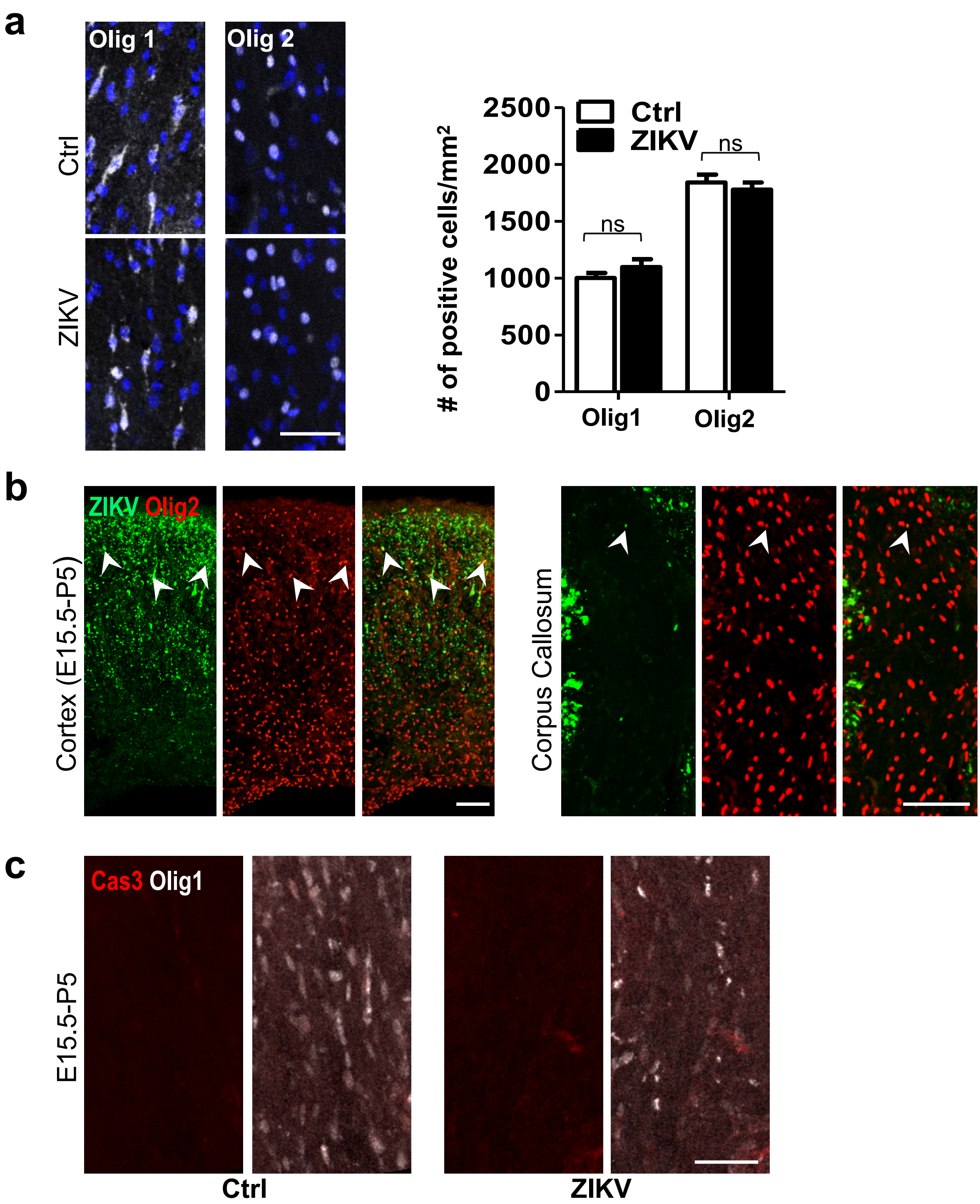

Figure S8. ZIKV infects OPCs and unlikely leads to apoptosis in Corpus Callosum. Coronal sections of neonate pups infected or mock-infected at E15.5 and inspected at P0 or P5. **(a)** Coronal sections of P0 pups were stained for Olig1 and Olig2. Right panel: quantification of Olig1 and Olig2 positive cells. Olig1: n=7/4, p=0.2538; Olig2: n=8/3 p=0.5137. **(b)** Coronal sections of infected pups were stained for ZIKV and Olig2. **(c)** Corpus Callosum regions were stained for the activated form of caspase 3 (Cas3) and Olig1. Data are mean ± SEM. ns: no significant. Scale bars: 100μm (b), 40μm (a,c).

Figure S9

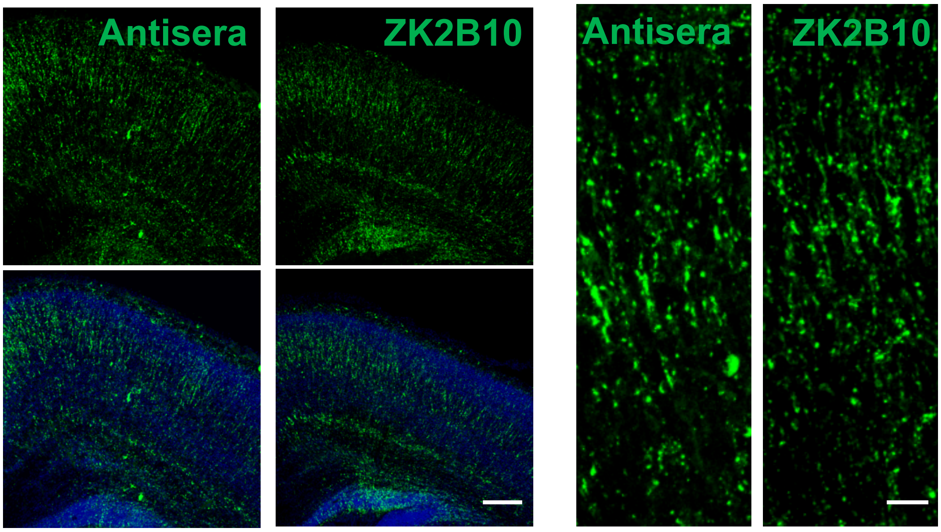

Figure S9. ZIKV antisera shows similar pattern with HmAb of ZIKV. Coronal sections of infected P0 were stained for ZIKV antisera and HmAb ZK2B10. Scale bars: 200μm (left panel), 40μm (right panel).

Figure S10

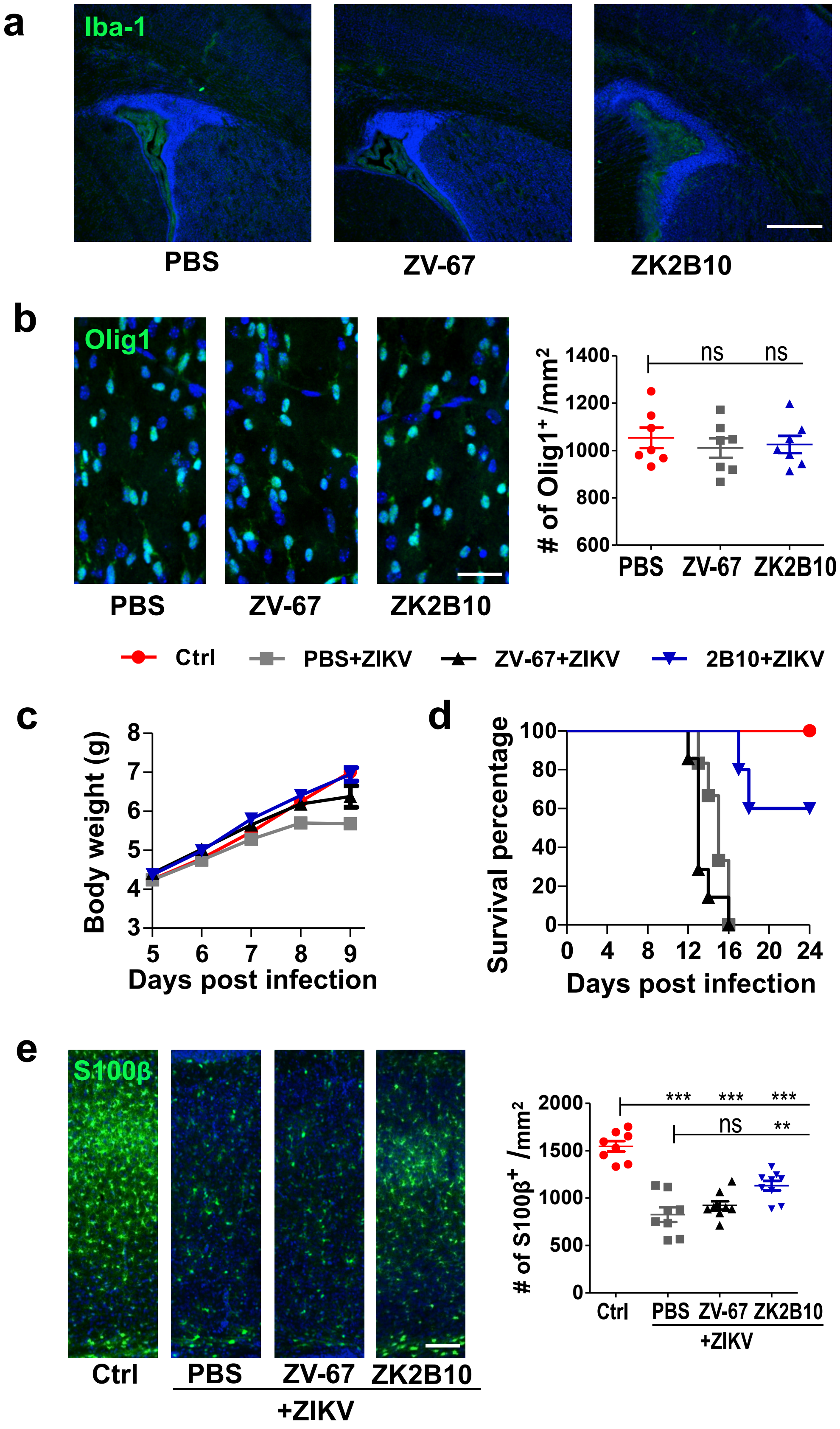
a
Iba-1
PBS
ZV-67
ZK2B10
b
Olig1
PBS
ZV-67
ZK2B10
# of Olig1+ /mm2
1400
1200
1000
800
600
ns
ns
PBS
ZV-67
ZK2B10
Ctrl
PBS+ZIKV
ZV-67+ZIKV
2B10+ZIKV
c
Body weight (g)
8
7
6
5
4
3
5
6
7
8
9
Days post infection
d
Survival percentage
100
80
60
40
20
0
0
4
8
12
16
20
24
Days post infection
e
S100β
Ctrl
PBS
ZV-67
ZK2B10
+ZIKV
# of S100β+ /mm2
2000
1500
1000
500
0
***
***
***
ns
**
Ctrl
PBS
ZV-67
ZK2B10
+ZIKV

Figure S10. The disruption of oligodendrocyte development by ZIKV can be prevented by ZK2B10. (a, b) Neonatal brains infected or mock-infected at E15.5 and inspected at P3. ZV-67, ZK2B10 or PBS was injected about 4-6h before virus injection. Slices of uninfected brains were stained with Iba-1 or Olig1. Quantified at right panel (b). n=7/3. PBS & ZK2B10: p=0.4865; PBS & ZV-67: p=0.6286. **(c-e)** Antibody or PBS was injected about 0.5h before virus or medium injection at P0. **(c)** Body weight of PBS, ZV-67 and 2B10 treated group mice from P5 to P9. Ctrl n=3, PBS+ZIKV n=6, ZV-67+ZIKV n=6, ZK2B10 n=8. **(d)** Survival percentage of each treated group. Ctrl n=6, PBS+ZIKV n=6, ZV-67+ZIKV n=7, ZK2B10+ZIKV n=5. **(e)** Slices from P9 brains were stained for s100β. Right panel: quantification of S100β^{+} cells. Ctrl n=8/3, PBS+ZIKV n=8/3, ZV-67+ZIKV n=9/3, ZK2B10 n=9/3. Ctrl & PBS+ZIKV: $p=2.6\times10^{-6}$; Ctrl & ZV-67+ZIKV: $p=2.1\times10^{-7}$; Ctrl & ZK2B10+ZIKV: $p=5.1\times10^{-5}$; PBS+ZIKV & ZV-67+ZIKV: p=0.2809; PBS+ZIKV & ZK2B10+ZIKV: p=0.0041. Data are mean ± SEM. ns: no significant, **p<0.01, ***P<0.001. Scale bars: 300μm (a), 40μm (b), 100μm (e).
